# Explore pre-hospital emergency challenges in the face of the COVID-19 pandemic: A quality content analysis in the Iranian context

**DOI:** 10.3389/fpubh.2022.864019

**Published:** 2022-08-17

**Authors:** Marziye Hadian, Alireza Jabbari, Mahdieh Abdollahi, Elaheh Hosseini, Hojjat Sheikhbardsiri

**Affiliations:** ^1^Health Services Management, Student Research Committee of School of Management and Medical Information, Health Management and Economics Research Center, Isfahan University of Medical Sciences, Isfahan, Iran; ^2^Health Services Management, Health Management and Economics Research Center, Isfahan University of Medical Sciences, Isfahan, Iran; ^3^Department of Nursing, Zarand Branch, Islamic Azad University, Zarand, Iran; ^4^Health in Disasters and Emergencies Research Center, Institute for Futures Studies in Health, Kerman University of Medical Sciences, Kerman, Iran

**Keywords:** pre-hospital emergency, challenges, COVID-19 pandemic, nurses, paramedic

## Abstract

**Background:**

pre-hospital emergency is a community-oriented system that responds to the medical needs of the injured or patients with acute and emergency illnesses outside of health care facilities until they are transferred to a medical center. This study aimed to explore pre-hospital emergency challenges in the face of the COVID-19 pandemic.

**Material and methods:**

This study was conducted as a qualitative content analysis in Iran. Using the purposive sampling method, data were collected through in-depth individual interviews with 28 prehospital paramedic personnel from November 2020 to November 2021. Graneheim and Lundman's conventional content analysis methods were used to analyze the data and for the trustworthiness of the data, this study used Lincoln and Guba's recommendations.

**Results:**

After multiple rounds of analyzing and summarizing the data and taking into consideration similarities and differences, four main categories and 10 subcategories were created based on the results of the data analysis and including (1) Culture and Community. (2) Service delivery (3) Human resources; (4) Medical supplies and equipment.

**Conclusion:**

According to the findings of this study emergency medical system employees are suffering from a range of psychiatric problems as a result of a lack of equipment and job overload, which has a detrimental impact on the quality of pre-hospital emergency care. Therefore, emergency care senior management should develop comprehensive guidelines, provide more equipment and minimize professional challenges to improve the quality and safety of pre-hospital emergency care services.

## Introduction

The COVID-19 outbreak originated in Wuhan, China, and has swiftly spread worldwide within a short period (1, 2). The COVID-19 outbreak is one of the most serious health threats, and it is likely to be the worst pandemic since 1918 (1, 2). Until April 19th, 2021, the total number of people infected with COVID-19 exceeded 142 million, and more than 3 million died of it. Iran, like other countries, is affected by the disease, and statistics show that it is one of the ten countries with the highest number of patients and deaths (3–6). A pre-hospital emergency is a community-oriented system that responds to the medical needs of the injured or patients with acute and emergency illnesses outside of health care facilities until they are transferred to a medical cent (6, 7). This system is engaged in responding to emergency telephone calls, visiting the scene, providing care by trained staff at the scene, continuing to provide care in vehicles such as ambulances and helicopters, and transferring the injured or victims to a medical center designated by the emergency operations center (2). As a result, pre-hospital emergency care plays a vital role in sustaining human life (8). Emergency medical services (EMS) also play a major role in natural disaster relief operations, including response to epidemics, and the role of EMS personnel as the first responders to biological disasters is well-documented (9).

Pre-hospital emergency has suffered many problems in recent decades, which have intensified in the face of hazards such as the COVID-19 pandemic, but it needs to be empowered to adapt to new changes and provide effective and quick responses to emergencies (10). The COVID-19 pandemic poses significant challenges to emergency care services, both in the community and in clinical and hospital settings (11). During the current COVID-19 pandemic, emergency medical service organizations around the world have faced this pandemic, while continuing to operate their pre-hospital routines (12). Routine work was affected both by the need for EMS staff to take precautions such as personal protective equipment (PPE), case overload, and the risk of transmission to themselves or their patients and also by patients' fear of transmission (3, 9).

Fever and respiratory problems in patients suspected of having COVID-19 originally disrupted prehospital care, forcing the EMS to modify its procedures (13, 14). In Tehran, EMS established a coronavirus monitoring committee, expanded the number of employees to properly respond to the increase in the number of patients, and initiated formal staff training to be able to adequately care for patients (7, 13). Iran's EMS Organization as one of the agile organizations of the Ministry of Health, Treatment and Medical Education and also as the first line of this system, has used all its power and capacity to provide valuable measures in the management of COVID-19 disease (15).

In Iran, Afzali et al. investigated the lessons acquired in a pre-hospital emergency system in Yazd during the COVID-19 pandemic, they concluded that organizations can use the lessons learnt from COVID-19 management to better examine their performance, methodically record and analyze concerns and challenges and deal with procedures to avoid future performance risks (15). Researchers in Canada investigated the impact of COVID-19 on emergency medical service call volumes and patient acuity and discovered that the virus may have caused actual changes in emergency medical service demand, which will be of interest to other services preparing for future pandemics or COVID-19 waves (16). Another study in the United States looked at quantifying changes in emergency medical services (EMS) occurrences as the COVID-19 pandemic expanded across the country, and discovered that there was a large decrease in the number of EMS responses across the country early in the outbreak. Simultaneously, the number of deaths attended by EMS doubled, but the number of injuries fell (17). Jensen et al. (18) investigated the call volume and the two methods put in place to deal with the increased call volume to the Copenhagen EMS, and their findings were presented. The emergency medical dispatch facility in Copenhagen was severely overburdened during the first month of the COVID-19 pandemic in Denmark, resulting in long lines and limiting citizens' access to emergency help and triage (18).

Pre-hospital emergency as the first line of care and treatment in dealing with out-of-hospital emergency patients is of considerable importance in the health care system (19). Due to the high workload and limited workforce, it is essential to identify challenges related to pre-hospital emergencies as a front line in dealing with critically ill patients in the management of the new coronavirus (COVID-19) pandemic in the country's medical universities. The identification of such challenges can contribute to developing a roadmap for future pandemic management planning. In this study, a qualitative method was used. Qualitative research involves the collection and analysis of non-numerical data (such as text, video, or audio) to understand concepts, opinions, or experiences (5, 20, 21). Qualitative research can be used to gather in-depth insights into a problem or to come up with new ideas for research (4, 5, 22). For this reason, in this study, to deeply identify the challenges and the applicability of the results for policymakers, the qualitative research method was used, to provide accurate and principled solutions. According to a survey of the literature, few qualitative studies have addressed the issues experienced by EMS personnel during the COVID-19 Pandemic. It will be impossible to improve the quality of prehospital emergency care without an awareness of emergency care personnel's extensive experience with the clinical issues of the COVID-19 pandemic. Understanding the thoughts and worries of EMS personnel in the aftermath of an infectious or contagious disease pandemic, such as the current COVID-19, can aid in the development of effective disaster management methods and improve the quality of pre-hospital emergency care. Thus, considering the importance of this issue this qualitative study aimed to explore pre-hospital emergency challenges in the face of the COVID-19 pandemic.

## Methods

### Design

This qualitative study was conducted from November 2020 to November 2021.The content analysis methodologies were applied in this qualitative study, which took place.

### Participants

This qualitative study took place in Iran, with 15 nurses and 13 pre-hospital paramedic workers as participants. A total of 16 men and 12 women between the ages of 21 and 52 were interviewed. A purposeful sampling strategy was used to choose the participants. Participants having a bachelor's degree in nursing or an associate's or bachelor's degree in prehospital emergency nursing, at least 1 year of work experience in a prehospital emergency center, and willingness to participate in the study met the inclusion criteria. The lack of consent to participate in the study and employees who have not had direct contact with patients with COVID-19 were exclusion criteria. Sampling continued until data saturation was reached, at which point the researcher judged that more interviews would not yield new information (23). Participants in this study were selected from centers that have been in direct contact with COVID-19 patients. Participants were also purposefully selected from all provinces of Iran country and data collection continued until data saturation.

### Data collection

In-depth interviews were used to conduct this qualitative study, which began with open questions and progressed to more specific questions, Showed in Table 1. Before the start of each interview, the research team talked about the importance of this study. Also, the research conditions were described and advice on how researchers conduct interviews that do not endanger their health or the health of other participants was sought. The interviews were recorded and lasted somewhere between 25 and 74 min. The time and place of the interviews were set by agreement between the researcher and the participants. Depending on the time of the interviews, the interviews are conducted in person in metropolitan areas and absentia in other provinces and by telephone. During the interviews, field notes were made to accurately describe and understand the responses.

**Table 1:** Interview questions for explore pre-hospital emergency challenges in the face of the COVID-19 pandemic.

1:“Please detail your experiences with COVID-19 pandemic against patients?”
2: The interviewers asked. “What were the most common causes of these issues and shortfalls for pre-hospital emergency service providers?”
3: “What were the most critical challenges and deficiencies for pre-hospital emergency service providers?”
4: “Would you clarify more?”

### Data analysis

The data acquired in this step was examined using Graneheim and Lundman's method of content analysis (24). The researcher examines the transcribed materials multiple times to fully comprehend them in this inductive approach. Then, the meaning units (words, sentences, or paragraphs) that responded to questions about pre-hospital emergency challenges and issues in handling the COVID-19 pandemic were identified, reduced, and coded. Similar codes were separated into subcategories, which were then concatenated to produce a category (manifest level). Each category arose from a collection of related ideas, making the categories internally homogeneous yet externally heterogeneous. The key subject emerged as the interaction between the underlying meanings in categories, which express the latent meaning. The interviews were conducted by E.H and HSH individually. The process of data collection was under the supervision of D.A.J.The MAX QDA 16 Software was used to handle the coding process.

### Trustworthiness

For reliability and validity tests, this study used Lincoln and Guba's recommendations (25). Lincoln and Guba evaluation method for validation of qualitative research is based on four axes validity, verifiability, reproducibility and provability. These criteria are widely used in the validation of qualitative research. For this reason, in internal studies, these two researchers are better known for the issue of validity and reliability in qualitative research. To ensure reliability, four requirements of creditability, dependency, conformability, and transferability are required, according to this suggestion. The researchers spent 11 months interacting with data and the environment, constantly making observations and compiling field notes, to improve data reliability. Peer check procedures were used to determine the data's dependability. Monthly peer checks were conducted to ensure that the study team had a full discussion of the newly discovered data. Background information and the researchers' interests in the respective topics, as well as document upkeep, were used to assess data conformability. The context of the interviews, codes and extracted categories were analyzed by the research team, as well as other experts and three professionals in the field of qualitative research. Using maximum variation sampling, the researchers were able to collect a diverse range of comments, observations, and interpretations.

### Ethical considerations

This study was authorized by the Isfahan University of Medical Sciences Ethics Committee. All methods were carried out in compliance with the applicable standards and regulations, and no animal research was carried out by any of the authors. Individual participants in the study gave their written informed permission. The participants' confidentiality and identities were maintained by the questioners' coding. Participants in the study were made aware of their right to withdraw from the study at any time without having to defend their decision.

## Results

### Demographic characteristics of the participants

There were 28 nurses and paramedic technicians in the study, with an average age of 36.5 ± 4.8 years and a range of 21–52 years. Their average work experience was 12.25 ± 3.4 years, and all of the participants had worked in EMS for more than 2 years, Showed in Table 2.

**Table 2:** Demographic information of participants.

**Age**	**Organizational position**	**Work experience**
41	Nurse	12
28	Nurse	4
45	Nurse	13
47	Nurse	12
46	Nurse	12
25	Nurse	12
51	Nurse	13
46	Nurse	15
29	Nurse	8
43	Nurse	15
41	Nurse	9
41	Nurse	7
42	Nurse	7
45	Nurse	11
31	Nurse	8
45	Manager	15
52	Manager	23
46	Manager	12
46	Manager	7
45	Technician	8
44	Technician	11
45	Technician	15
47	Technician	10
46	Technician	8
39	Technician	20
50	Technician	10
43	Technician	18
35	Technician	14

## Main results

After multiple rounds of analyzing and summarizing the data and taking into consideration similarities and differences, four main categories and 10 subcategories were created based on the results of the data analysis and including (1) Culture and Community. (2) Service delivery (3) Human resources; (4) Medical supplies and equipment, as shown in Table 3. The categories and corresponding sub-categories are described in the following sections.

**Table 3:** The Categories, sub-categories and codes extracted from the data.

**Categories**	**sub-categories**	**Example code**
Culture and community	Public immorality	Unnecessary calls from people in the community
		Providing untrue histories by people to EMS staff and wasting their time
		Improper treatment of patients
	Lack of public awareness	Lack of public knowledge and awareness about the symptoms of the disease the occupation of emergency telephone lines and waste of staff time
		Increased demand and calls due to people's anxiety and worry
		Not using other telephone numbers introduced for consultation
Service delivery	Procedural challenges	The absence of any protocol for patients with cardiac trauma
		Ineffective allocation of resources due to lack of accurate registration of patient information in the MCMC system
		The absence of a referral system for COVID-19 patients
		Unfair distribution of personal protective equipment (PPE)
	Infrastructural challenges	Lack of a mechanism for registering COVID-19 patients in the crisis care system
		Ineffective distribution of pre-hospital emergency services especially in rural and remote areas
Human resources	Quality	Hiring inexperienced staff
		Hiring non-specialist health workers
		Lack of financial incentives
	Training and skills	Insufficient training for disinfecting ambulances
		Lack of specialized training courses for EMS personnel dealing with COVID-19 patients
	EMS staff's health	Psychological distress
		Burnout
	Safety and health	Lack of standard personal protective equipment (PPE)
		Non-observance of health protocols by some staff
Medical equipment and supplies	Physical resources	Lack of special personal protective equipment (PPE)
		Inadequate equipment in ambulances
		Lack of specialized and coded ambulances
	Financial resources	Inadequate financial support for EMS
		Lack of financial resources to provide personal protective equipment (PPE)

## Cultural and social factors involved in poor management of the COVID-19 pandemic in EMS

Analysis of the interviews showed that most of the participants stated that Unnecessary calls from people in the community providing untrue histories by people to EMS staff and wasting their time, improper treatment of patients, lack of public knowledge and awareness about the symptoms of the disease, and the occupation of emergency telephone lines and waste of staff time, increased demand and calls due to people's anxiety and worry, and not using other telephone numbers introduced for consultation were some social and cultural challenges faced by the EMS center in dealing with the COVID-19 pandemic. These challenges were divided into two categories of public immorality and public unawareness.

### Public immorality

The various ethics and morality issues stemming from the pandemic's public policy challenges, particularly as it relates to medical care decision-making and the fairness of measures established to contain the pandemic and care for its victims, are one of the key concerns. According to one of the participants:

“*One of the challenges is some people play a trick on EMS staff. I hear from kids that they have called 115 emergency phone numbers and reported faked COVID-19 symptoms and wasted the time and energy of the staff. These pranks make our phone line busy and real patients find problems contacting us*”. “*More than 50% of calls to EMS are prank calls. These unwarranted calls reporting untrue symptoms waste a lot of time of EMS staff, constantly occupying the lines and people simulating symptoms. Sometimes they call and report unrealistic symptoms to know true symptoms and to see what the staff's reaction is !!!!*” *(Participant 2)*.

### Lack of public awareness

lack of public knowledge, widespread panic and worry among the general community due to an unknown sickness, as well as poor health facilities, provide particular obstacles and a significant threat to the population. One of the participants stated that:

“*People are not cooperative and they occupy 115 EMS hotlines for no reason. Besides, the non-use of other telephone numbers introduced by the Ministry of Health to answer questions related to COVID-19 has become very troublesome. In general, some measures should be taken so that people resort to self-quarantine at home due to inconsiderate behaviour of some individuals*”. “*Another problem is the frequent contact made by people due to confusion and lack of knowledge about the symptoms of the disease. They call the center even with a cough or sneeze and have lots of anxiety and stress that they are infected with COVID-19*” *(Participant 13)*.

## Service delivery related factors involved in poor management of the COVID-19 pandemic in EMS

Most of the participants stated that the absence of any protocol for patients with cardiac trauma, ineffective allocation of resources due to lack of accurate registration of patient information in the MCMC system, the absence of a referral system for COVID-19 patients, unfair distribution of personal protective equipment (PPE), lack of a mechanism for registering COVID-19 patients in the crisis care system, and ineffective distribution of pre-hospital emergency centers, especially in rural and remote areas, were the most important challenges of providing hospital emergency services in the face of the COVID-19 pandemic. These challenges were divided into two categories: procedural and infrastructural challenges:

### Procedural challenges

In the COVID-19 pandemic, a better understanding of the obstacles faced by EMS staff could aid in enhancing the quality of pre-hospital emergency care. According to one of the participants:

“*The main problem faced by EMS staff is that they don't know exactly what to do for the patient. They do not have enough protective equipment. We do not know how to transfer patients to the hospital and how to treat them. For example, some patients call EMS for heart problems but the staff don't know what to do. Thus, we must have some instructions for these cases*”. “*It's very important to accurately record the information of COVID-19 patients in the MCMC system of the Ministry of Health because many resources assigned under the Foreign Exchange Savings Board Law by the Ministry of Health must be distributed based on statistics and information of this system presented to medical universities but unfortunately this isn't done currently*” *(Participant 9)*.

## Infrastructural challenges

It will be important to re-assess the approach toward testing oversight and implement changes that will focus on regulatory adaptability and developing the infrastructure needed to efficiently respond in the time of such crises. One of the participants stated that:

“*Given the failure in rapid and accurate diagnosis of suspicious COVID-19 cases and the absence of a mechanism to register and report these cases in the crisis care system, many indicators, and statistics related to COVID-19 patients are not provided to medical universities accurately*”. “*The shortage of EMS staff and ineffective distribution of emergency pre-hospital centres, especially in remote rural areas is a major challenge that the EMS center faces and needs to be addressed as soon as possible*” *(Participant 22)*.

## Workforce-related factors in poor management of the COVID-19 pandemic in EMS

The findings indicated most of the challenges identified in this study were related to the EMS workforce. A majority of the participants referred to hiring inexperienced staff, hiring non-specialist health workers, lack of financial incentives and rewards, insufficient training for disinfecting ambulances, lack of specialized training courses for EMS personnel dealing with COVID-19 patients, psychological distress, burnout, lack of standard personal protective equipment (PPE), non-observance of health protocols by some staff as the main manpower challenges during the COVID-19 pandemic in the pre-hospital emergency department. These challenges were divided into four categories: Quality, training and skills, EMS staff's health, and safety and health.

### Quality

Understanding the thoughts and worries of EMS personnel in the aftermath of an infectious or contagious disease pandemic, such as the current COVID-19, can aid in the development of effective disaster management methods and improve the quality of pre-hospital emergency care. According to one of the participants:

“*Unfortunately, the staff with more service records and experience often take leave due to fear of developing the disease and thus the workload is taken by newly employed or plan-based staff who have no or little experience and are not permitted to take a leave of absence. This lowers the quality of EMS services offered and leads to fatigue and burnout among the staff* ”. “*Due to the shortage of manpower in the emergency center, the staff hired in the center do not have any expertise and knowledge in this field. Sometimes, the staff from other units are used in the center and the lack of necessary knowledge and training in the staff only causes rework and a higher risk of COVID-19 transmission*” *(Participant 4)*.

### Training and skills

The proper training on how to utilize personal protective equipment (PPE) to protect EMS personnel from infectious diseases is crucial to keeping a stable workforce ready for COVID-19 outbreaks. During the COVID-19 pandemic, there have been few descriptions of EMS personnel's perceptions on PPE knowledge, use, and training. One of the participants stated that:

“*Due to the excessive bleach, the equipment has been severely damaged. The lack of knowledge and training necessary for the proper disinfection of ambulance spaces by the EMS staff has caused a lot of damage to the equipment*”. “*Given the shortage of workforce, newly-hired staffs start working without any training. as they don't have adequate knowledge of the concepts and instructions, they may commit errors while performing their tasks and sometimes they become infected with the coronavirus due to mistakes in the work processes. “Everyone from the top levels of management to the pre-hospital staff does not know how to deal with COVID-19 patients due to low awareness and lack of facilities. It would have been better if the Ministry of Health had set up some training courses for all of them*” *(Participant 9)*.

## EMS staff's health

According to the participants in this study, EMS personnel's occupational health in the face of the coronavirus is mostly dependent on their ability to obtain adequate PPE. According to one of the participants:

“*Unfortunately, EMS staff are suffering from a lot of burnout. In addition to traffic and urban congestion, they work 24 hours a day and they may get burnout. In my opinion, the ministry must put some plans into action to supply an adequate workforce for places such as inpatient and emergency departments. This helps the staff remain in good condition. The staff also need financial support, incentives, etc., so that they can stand these difficult conditions, but unfortunately, such plans are currently nonexistent*” *(Participant 3)*.

## Safety and health

According to the participants, a consistent scientific approach is required for a proper evaluation of EMS personnel's performance in caring for COVID-19 patients in order to assure high-quality care and the safety of these patients. One of the participants stated that:

“*Standard personal protective equipment is not adequately delivered to staff and this has made the drivers get the COVID-19 disease and also easily transmit the disease to others, and thus leading to the failure in breaking the chain of transmission*”. “*In some cases, the staff do not comply with the health protocols and do not pay any attention to what their colleagues' advice. This puts both other staff and their families at risk. In addition, other staff working with these people undergo a lot of stress. The situation can get better by imposing fines on these people and forcing them to follow health protocols*” *(Participant 27)*.

## Resource-related factors in poor management of the COVID-19 pandemic in EMS

Analysis of the participants' interviews indicated that a majority of them considered the lack of special personal protective equipment (PPE), inadequate equipment in ambulances, lack of specialized and coded ambulances, inadequate financial support for EMS, and lack of financial resources to provide personal protective equipment (PPE) were the most important challenges faced by the EMS center in managing COVID-19. The resource-related challenges are divided into two categories: Physical and financial resources:

### Physical resources

Healthcare administrators should conduct regular demand analyses and allocate resources based on the number of healthcare providers and the expected patient volume. According to one of the participants:

“*The centre suffers from a lack of specialized and coded ambulances to transport suspicious patients to medical centers. Ambulance drivers do not follow the special protocols for dealing with COVID-19 patients and there is not adequate personal protective equipment according to related standards. This has caused both drivers to become infected with COVID-19, and the patient transfer chain at this hospital has been disrupted*”. “*The EMS staffs usually go to extremes when using personal protective equipment. They wear full protective equipment when transferring COVID-19 patients similar to movies and with a state of pride and showoff and sometimes getting carried away! But when transferring ordinary patients, they don't even were simple masks or gloves. Sometimes when taking a CT scan of the chest to diagnose the degree of fracture, the doctor notices a coronavirus infection in the bottom of the lung, which is very normal as the patient has been contacting lots of people including pre-hospital emergency staff unprotected after having an accident*” *(Participant 6)*.

### Financial resources

Financial performance as a metric for determining how well a corporation manages its financial resources. One of the participants stated that:

“*Due to insufficient funds allocated to this center, the triage nurses and staff in this center do not have enough medical equipment except a thermometer and oxygen meter, and there is not enough personal protective equipment in the triage division of this center*”. “*Not allocating enough funds to provide personal protective equipment in pre-hospital emergency centers has become a major problem. Really, given that we are at the forefront of the fight against COVID-19 and dealing directly with patients, we should not face such stresses and challenges*”. *(Participant 14)*.

## Discussion

The present study aimed to identify the pre-hospital emergency challenges in the face of the COVID-19 pandemic. Based on the findings of the study, four categories and 10 sub-categories related to pre-hospital emergency challenges during the COVID-19 pandemic were extracted, as discussed below:

### Category 1: Culture and community

A majority of the participants in the study pointed to the challenges of increasing demand and calls to the EMS center due to public anxiety. Public anxiety and worry in such epidemics are normal but will cause problems in providing pre-hospital services. Al Amiry et al. (9) suggested that during the development of COVID-19, there was a global phenomenon of exponential increase in EMS calls. In New York City alone, for example, the volume of EMS calls increased from the usual daily high of 4,000 to more than 7,000, and this dramatic increase in call volume is expected to put a lot of pressure on EMS centers, as indicated in the present (9). According to research by Ferron et al. during the first 5 months of the COVID-19 outbreak, however, EMS calls in the Niagara area declined dramatically (16) which was inconsistent with the results of the present study. Nevertheless, the massive influx of call volumes requires EMS systems to re-examine their IT infrastructure and personnel to increase their call capacity (9).

### Category 2: Service delivery

Concerning service delivery challenges in providing services, the participants believed that there was no fair distribution of personal protective equipment. A similar study by Maguire et al. (26) suggested that challenges for EMS staff may be more difficult because they are one of the groups most likely to be affected by COVID-19. Thus, having proper PPE affects their performance (26). Another service delivery challenge highlighted in the present study was the lack of protocols for patients. As a result, Mohammadi et al. emphasized the importance of developing a thorough and systematic pre-hospital care procedure for EMS staff (27). The findings of this study concerning infrastructural challenges in service delivery highlighted the inadequacy of specialized and coded ambulances. Similarly, One of the significant challenges in the correct handling of the COVID-19 pandemic in pre-hospital emergency care, according to Mohammadi et al. (27). is the lack of ambulances specifically tailored for COVID-19 cases

### Category 3: Human resources

The outcomes of this study reveal that one of the issues of pre-hospital emergency services is EMS worker stress and burnout during the current COVID-19 pandemic. Healthcare management, according to Parvaresh-Masoud et al. (28), should take steps to guarantee the psychological safety of EMS staff and prevent burnout. Because these factors have the potential to influence the quality of care (28). The lack of personnel training and competence was cited as a significant barrier to providing EMS services by the participants in this study. Cash et al. found that despite their education, 40% of EMS professionals in the United States still need to be trained in the N95 fit testing process (29).

The participants in the present study believed that fear of developing the disease and involvement with it in the workforce was one of the challenges in providing pre-hospital services. Similarly, Labaf et al. showed that the fear of COVID-19 in medical and hospital staff due to their direct exposure to patients or its transmission to others and family members is one of the most important human resource challenges (30). Furthermore, (31) referred to the presence of moderate to severe depression and anxiety in more than half of the pre-hospital emergency staff and stated that EMS staff play an important role in improving and promoting the health of people in the community, eliminating the underlying factor, causing emotional reactions in them should be considered a health priority. In addition, Siman-Tov et al. (32) believed that extreme concerns about transmission of the disease made medical staff fearful of transporting patients to the hospital in an emergency. This is also a real concern because patients with critical conditions that showed obvious signs of deterioration due to negligence admitted that they had delayed seeing a doctor for fear of spreading COVID-19 in the hospital (21, 32).

Khankeh et al. Also mentioned the challenge of nurses' burnout along with constant stress, insufficient support, inability to manage human resources and disregard for their needs and stressed that due to long working hours and multiple increases in the number of patients hospitalized. And high workload has reduced the mental strength and physical, emotional and mental fatigue of health care workers (33). Therefore, due to the increase in the number of hospitalized patients and the extensive workload of these employees and their frustration, it requires better management. Findings from the study of Garosi et al. (34) also show that many psychosomatic problems have affected nurses. Physical and mental fatigue, skin problems, chronic headaches, and anxiety disorders are some of these, many of which have been cited and accounted for a significant volume of studies (34).

The issue of “manpower shortage” was another challenge mentioned by the participants. Labaf et al. also emphasized the challenge in their study by expressing the shortage of specialized manpower (35). In this regard, Garosi et al. Also mentioned the lack of specialized and experienced nurses, unconventional work schedules, involuntary transfer of power between departments, insufficient support of the organization and insufficient specific training for Coronavirus as important problems for nurses in this period (34).

Interviewees pointed to the lack of equitable distribution of personal protective equipment, and Labaf et al. (35) noted that there is a challenge in controlling the consumption of protective equipment concerning important management challenges. There is enough equipment, but there is no control over their consumption and distribution (35).

“Poor training of medical staff” is also an important challenge, and neglecting this issue leads to insufficient knowledge and hinders their ability to work safely. Similar studies such as Sun et al. (36), Yin and Zeng (37), and (38) report the importance of nursing education during the Corona epidemic. Lack of awareness is one of the main causes of insecurity and providing training in the prevention and control of COVID-19 can also reduce the psychological burden and insecurity of nurses (36, 37).

### Category 4: Medical equipment and supplies

One of the challenges highlighted by the participants in this study was the lack of medical equipment and supplies. A similar study by Al Amiry et al. showed that worldwide quarantine disrupted the global supply chain, including the medical sector, leading to a shortage of medical resources and equipment, and significantly affecting countries with poor health infrastructure and limited resources (9). The findings of the Razu study show that incentives such as financial support, continuous monitoring, adequate protective equipment and adequate manpower can encourage health workers to be more involved in epidemic conditions (39). Another important difficulty affecting the care offered to COVID-19 patients in pre-hospital emergency care, according to the participants, was the unavailability of special personal protective equipment and inadequate personal protective equipment allocation. (40) also pointed out the inadequacy of specific personal protective equipment, as was supported in the present study. Thus, it is essential to allocate personal protective equipment fairly and effectively.

## Limitations and strengths of the research

This study has a strong point considering that it has been done in the whole country and its challenge has been extracted all over the country. However, due to the prevalence of Quaid disease, some interviews were not possible in person and had to be conducted virtually.

## Conclusion

Raising public awareness about COVID-19 and how to manage it can prevent many injuries. During a pandemic, team awareness and remote physician support may reduce unnecessary referrals and minimize mortality and complications. Furthermore, conducting training programs aimed at optimizing available resources is a low-cost, high-impact intervention strategy to strengthen pre-hospital care systems. According to the findings of this study, EMS employees are suffering from a range of psychiatric problems as a result of a lack of equipment and job overload, which has a detrimental impact on the quality of pre-hospital emergency care they provide during the current health pandemic. Protecting the mental health of employees working with COVID-19 patients should be one of the top considerations for medical facility directors in these circumstances. Furthermore, during the present COVID-19 pandemic, emergency care senior management should develop comprehensive guidelines, provide more equipment, and minimize professional challenges to improve the quality and safety of pre-hospital emergency care services.

## Data availability statement

The original contributions presented in the study are included in the article/supplementary material, further inquiries can be directed to the corresponding author/s.

## Ethics statement

The studies involving human participants were reviewed and approved by Isfahan University of Medical Sciences. The patients/participants provided their written informed consent to participate in this study.

## Author contributions

The study's concept and design were created by MH. The survey was performed by AJ. Data analysis and manuscript writing were handled by HS, EH, and MA. AJ oversaw the research and provided critical feedback on the manuscript. The final manuscript was read and reviewed by all authors.

## Funding

This study was supported by Vice Chancellor for Research and Technology, Isfahan University of Medical Sciences, Isfahan, Iran (IR.MUI.NUREMA.REC.1400.205).

## Conflict of interest

The authors declare that the research was conducted in the absence of any commercial or financial relationships that could be construed as a potential conflict of interest.

## Publisher's note

All claims expressed in this article are solely those of the authors and do not necessarily represent those of their affiliated organizations, or those of the publisher, the editors and the reviewers. Any product that may be evaluated in this article, or claim that may be made by its manufacturer, is not guaranteed or endorsed by the publisher.
